# Update on new treatments for rare ovarian tumours

**DOI:** 10.1097/GCO.0000000000000836

**Published:** 2022-11-18

**Authors:** Stanislas Quesada, Marta Bini, Coriolan Lebreton, Isabelle Ray-Coquard

**Affiliations:** a Centre Léon Bérard, Lyon, France; b Institut régional du Cancer de Montpellier, Montpellier, France; c Instituto Nazionale dei Tumori, Milan, Italy; d Institut Bergonié, Bordeaux, France

**Keywords:** academic research, clinical practice, clinical trials, expert networks, rare gynaecological tumours

## Abstract

In spite of their rarity when considered individually, the sum of all rare ovarian tumours (ROT) represent almost half of all ovarian malignancies. As such, their appropriate inclusion within dedicated clinical trials is essential for enhanced management.

**Recent findings:**

Supported by institutional expert national (e.g. TMRG) and international (e.g. ESGO) networks and owing to national (e.g. ARCAGY-GINECO) and international (e.g. ENGOT) collaborations dedicated to clinical research, the last few years have shown increased number of clinical trials dedicated to ROT. These either were based on specific molecular features of ROT (e.g. expression of oestrogen receptors for low-grade serous ovarian carcinomas and anastrazole evaluation in the PARAGON trial) or on the evaluation of innovative therapies (e.g. pembrolizumab within the ROT cohort from the AcSé Pembrolizumab multicentric basket trial). Furthermore, recent years have also shown the advent of randomized clinical trials. For instance, the ALIENOR trial positioned weekly paclitaxel as a new option for relapsed sex cord-stromal tumours, while the GOG281/LOGS trial raised trametinib as a new standard-of-care option for recurrent low-grade serous carcinomas.

**Summary:**

The last few years have exhibited a paradigm shift towards the possibility to develop dedicated trials for ROT, owing to international collaborations supported by institutional networks. Current trials, molecular-driven and based on innovative designs, are highly promising, as they may bring ROT management towards more personalized medicine.

## INTRODUCTION

Rare cancers are defined by an incidence of less than 6 per 100 000 per year; conversely, they represent altogether over 20% of all diagnosed cancers in the European Union each year [1]. Following the same trend, while rare ovarian tumours (ROTs) exhibit limited incidences when taken as distinct entities, their sum nevertheless represents almost half of all ovarian malignancies [2]. In spite of substantial achievements regarding high-grade serous ovarian cancers (HGSOC) in the last 5 years, research regarding ROT have been limited until recently [3]. Indeed, ROT suffered from misclassification and limited treatment guidelines [4]. Owing to their rarity, ROT had been overlooked for a long time, and better knowledge and management of these entities have been possible through the development of dedicated consortia. At the national scale, two complementary institutional networks have been built: the *Tumeurs Malignes Rares Gynécologiques* (TMRG) network of expert centres dedicated to management of ROT and the *Association de Recherche sur les CAncers dont GYnécologiques Groupe d’Investigateurs National des Etudes des Cancers Ovariens et du sein* (ARCAGY-GINECO) supporting clinical research. At the European scale, a same construction can be observed with the European Society of Gynaecological Oncology (ESGO) and the European Network for Gynaecological Oncological Trial groups (ENGOT), respectively.

Until the past decade, the vast majority of ROTs were considered as a global entity, without harmonized treatment patterns; starting from empirical and expert-based management, a progressive paradigm shift has emerged [1,4,5]. Development of national and supranational consortia based on institutional collaborations led to substantial improvements regarding accurate classification, including systematic referral to histological review by pathologist experts and molecular characterization [6]. At the international scale, the Gynecologic Cancer Inter Group (GCIG) published a series of dedicated consensus reviews for ROT in 2014 [7–13]. Inclusion of ROT into randomized clinical trials (RCTs) was previously considered technically barely feasible [14]. Nevertheless, supported by aforementioned expert networks and international collaborations, the last 2 years have shown increased development of dedicated RCT and single-arm trials, leading to substantial clinical achievements with higher levels of evidence [15–17]. Recently, the sixth GCIG consensus guidelines have been published, highlighting the necessity of sustaining international multicentre trials with randomization against reference therapy; even for very rare subgroups, building of innovative designs such as platform studies was encouraged [18^▪^].

In this review, we will firstly discuss the recent results of the most relevant clinical trials regarding ROT and subsequently describe ongoing clinical trials and future perspectives. 

**Box 1:**
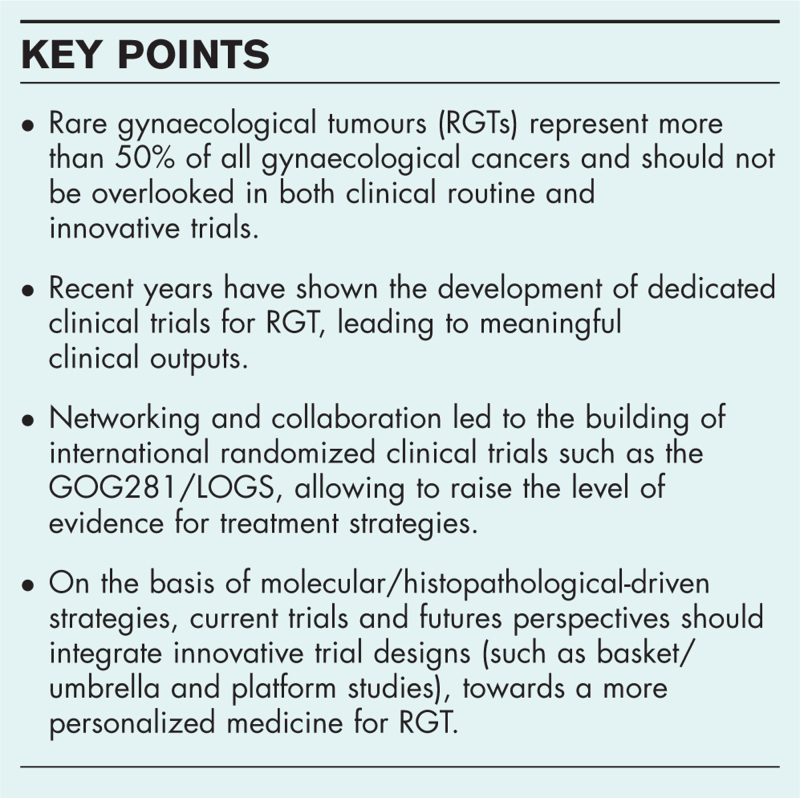
no caption available

## LOW-GRADE SEROUS OVARIAN CANCER

During the past decades, low-grade serous ovarian cancers (LGSOCs) were treated as HGSOC. Nevertheless, the overall response rate (ORR) to cytotoxic chemotherapy has been reported to be lower, with 23% and less than 5% in first-line and recurrent settings, respectively [19,20]. On the basis of retrospective data suggesting better endocrine therapy response in LGSOC, the prospective phase II PARAGON (ACTRN1261000796088) basket trial (LGSOC cohort) enrolled 36 patients with a recurrent LGSOC and evaluated anastrozole therapy; it reported that 63.9% of them did not progress at 3 months and a clinical benefit was still observed at 6 months [21]. Recently, the primary results (*n* = 15) of a phase II pilot study (NCT03531645), which evaluated abemaciclib and fulvestrant combination in the neoadjuvant setting for unresectable stage III/IV LGSOC, were reported, exhibiting substantial results with a clinical benefit rate of 80% (*n* = 12) [22]. Furthermore, interval cytoreductive surgery could be performed in five patients, with complete gross resection in four of them.

Regarding ongoing phase III clinical trials, the NRG-GY019 (NCT04095364) aims to evaluate adjuvant carboplatin-paclitaxel chemotherapy versus letrozole endocrine therapy in stage II-IV LGSOC [23]. Similarly, the international MATAO (NCT04111978) study compares letrozole versus placebo as maintenance after adjuvant chemotherapy [24]. Further questions remain, such as the optimal cut-off of endocrine receptor positivity and the relevance of endocrine therapies combinations, especially with CDK4-6 inhibitors. Thus, the phase II GOG-3026 (NCT03673124) study evaluates ribociclib and letrozole doublet in recurrent LGSOC [25].

Apart from oestrogen-related tumour driving, MAP kinase signalling pathway deregulation (through *KRAS, BRAF, NRAS* or *ERBB2* alterations) is a major contributor; indeed, it has been estimated to be present in roughly 60% of LGSOC [26–28]. Nevertheless, the MILO (NCT01849874) trial failed to demonstrate improved progression-free survival (PFS) with binimetinib (versus chemotherapy) in recurrent LGSOC [29]. Conversely, the recent results from the phase II/III GOG-281 (NCT02101788) trial positioned trametinib as potently new standard of care for recurrent LGSOC [30]. It randomized patients with recurrent LGSOC previously treated with at least one platinum-doublet regimen in two arms: trametinib versus standard of care treatment (chemotherapy or endocrine therapy). This trial met its primary endpoint, with a median PFS of 13.0 months [95% confidence interval (95% CI) 9.9–15.0) versus 7.2 months (95% CI 5.6–9.9) in the trametinib and standard-of-care groups, respectively (hazard ratio 0.48; 95% CI 0.36–0.64; *P* < 0.0001)].

Finally, immunotherapy could emerge as an interesting option for platinum-resistant LGSOC. The phase II, multicentric, AcSé pembrolizumab (NCT03012620) basket trial investigates the efficacy and safety of pembrolizumab in distinct rare tumours. A cohort specifically enrolled rare ovarian cancers (*n* = 62), including 23 LGSOC [31]. Interestingly, from the 21 patients with evaluable response, 11 exhibited clinical benefit from pembrolizumab.

## OVARIAN CLEAR CELL CARCINOMAS

Ovarian clear cell carcinoma (OCCCs) exhibit distinct clinical and molecular features when compared with other epithelial ovarian cancers, with a context of endometriosis found in 50–70% of the cases [32,33]. Furthermore, a higher incidence of OCCC is observed in the Eastern-Asian region [7]. In spite of more frequent early stage, chemosensitivity is low in OCCC, especially in platinum-resistant relapse, with an ORR of 1–33% [34]. Even in platinum-sensitive relapse, a partial response is observed in only 9% of the cases [35]. Hepatocyte nuclear factor-1β (HNF-1b) and vascular endothelial growth factor (VEGF) are commonly expressed, suggesting angiogenesis as a potential effective target in OCCC [36]. On the basis of data from the ICON7 (NCT00483782) and GOG-0218 (NCT00262847) studies, adjunction of bevacizumab to chemotherapy in primary treatment of OCCC has been used in clinical practice [37,38]. A monocentric retrospective study conducted in Japan assessed median PFS in stage III/IV OCCC, before and after bevacizumab approval in Japan in 2013 [39]. Median PFS was improved among the 18 patients treated with bevacizumab compared with 10 patients without (29.8 versus 12 months, *P* = 0.026). Recently, a retrospective study that used the same design but with a multicentre recruitment (with bevacizumab: 43 patients; without bevacizumab: 102 patients) reported an increase of median PFS (29.7 versus 12.5 months; *P* = 0.023) [40]. Furthermore, the median overall survival (OS) increased from 34.7 to 51.4 months (*P* = 0.085).

Recently, two single-arm, multicentre, phase II trials reported interesting data at the European Society for Medical Oncology (ESMO) congress 2022. The British PEACOCC (NCT03425565) study enrolled 49 recurrent clear cell carcinomas (of whom 85.4% were ovarian) and exhibited promising efficiency of pembrolizumab monotherapy, with a 12-week PFS rate of 43.8% (90% CI 31.5–56.6) [41]. Furthermore, the Chinese INOVA (NCT04735861) study investigated the potential benefit of combining sintilimab and bevacizumab for recurrent OCCC [42]. Preliminary results on 23 patients (of whom 18 were platinum-resistant and 20 with radiological evaluation) reported an interesting ORR of 40.0% (one complete and seven partial responses; 95% CI, 19.1–63.9).

Apart from antiangiogenetics and immunotherapy, OCCC could be targeted through its specific molecular features. Although *TP53* and *BRCA1/2* alterations are rare (13 and 6%, respectively), OCCCs exhibit *ARID1A* and *PIK3CA* mutations, which are found in roughly 50% of cases [43–45]. On the basis of these alterations, new trials are ongoing, such as the ATARI (NCT04065269) trial targeting *ARID1A* alterations with the ATR inhibitor ceralasertib [46]. According to the modest efficacy of conventional chemotherapies, biomarker-driven trials are of high concern for OCCC.

## OVARIAN SEX CORD-STROMAL TUMOURS

Ovarian sex cord-stromal tumours (OSCSTs) represent 7% of all ovarian malignancies and include adult or juvenile granulosa (70%) and Sertoli-Leydig cell tumours. Adult granulosa tumours harbour *FOLX2* mutations in 97% of cases [47]. Usually, OSCSTs are treated with surgery and adjuvant therapy can be considered in specific conditions, either with BEP (bleomycin, etoposide and cisplatin) or carboplatin-paclitaxel regimens [48]. Usually, these tumours are associated with a good prognosis but can recur in 20–25% of the cases. Different treatment options have demonstrated some efficacy, but there is no standard regimen. On the basis of their rich vasculature and their overexpression of VEGF, using antiangiogenics appeared as a relevant option for granulosa tumours [49]. Nevertheless, clinical data have given contrasted results. Indeed, a seminal phase II study (*n* = 36), which evaluated bevacizumab alone in recurrent OSCST demonstrated efficacy of this agent, with six (16.7%) and 28 (77.8%) patients exhibiting partial response and stable disease, respectively; furthermore, the median PFS was 9.3 months [50]. On the basis of these encouraging findings, the open-label, academic, international, randomized phase II ALIENOR (NCT01770301) trial evaluated weekly paclitaxel alone or in combination with Bevacizumab in relapsed OSCST [51]. Of the 60 patients enrolled, 32 received paclitaxel alone and 28 received combination treatment. Although the ORR was higher in the combination therapy arm (44%; 95% CI 26–65) compared with the paclitaxel alone arm (25%; 95% CI 12–43), median PFS appeared similar in both groups: 14.7 months (95% CI, 11.5–18.3) with single-agent paclitaxel versus 14.9 months (95% CI, 8.3–19.3) with combination therapy. Nevertheless, this study allowed to position weekly paclitaxel as a new option for recurrent OSCST. Regarding endocrine therapies, the OSCST arm from the PARAGON (ACTRN12610000796088) trial evaluated anastrozole in hormone receptor positive relapsed granulosa cell tumours [52]. The clinical benefit rate at 12 weeks based on 38 patients evaluated was 78.9% (*n *= 30, of whom one had partial response and 29 had stable diseases) and median PFS was 8.6 months (95% CI 5.5–13.5).

## CARCINOSARCOMAS

Carcinosarcomas, which represent less than 5% of all ovarian cancers, exhibit distinct features: preferably occurring in elderly, frequent late-stage diagnosis and aggressive diseases. At the molecular scale, they are characterized by a copy number high phenotype and *TP53* mutations in majority of cases [53]. Paclitaxel as well as ifosfamide is a commonly used regimen for sarcomas, including uterine carcinosarcomas [8]. Nevertheless, this regimen exhibits a particular toxicity profile and organizational constraints with 3 days of infusion; conversely, carboplatin along with paclitaxel globally leads to a better tolerance and allows an outpatient scheme. On the basis of these considerations, the NRG consortium constructed a phase III randomized trial (NCT00954174), which tested the null hypothesis that paclitaxel and carboplatin was inferior to paclitaxel and ifosfamide for the treatment of carcinosarcomas [54]. This study enrolled both uterine and ovarian carcinosarcomas (the latter being 90 patients). The study met its primary endpoint and demonstrated noninferiority of carboplatin along with paclitaxel combination. Noteworthy, among ovarian carcinosarcomas, those in the carboplatin and paclitaxel arm exhibited longer OS (30 versus 25 months) and PFS (15 versus 10 months) than those in the paclitaxel and ifosfamide arm, without reaching statistical significance. Apart from conventional cytotoxic molecules, the AcSé pembrolizumab (NCT03012620) basket trial gave poor signals regarding carcinosarcomas [31]. Indeed, although the clinical benefice rate in the whole cohort reached 44.6%, three out of the four patients with carcinosarcomas exhibited progressive disease and one had stable disease.

## PERSPECTIVES

Through the past decade, several improvements emerged regarding the management of ROT, mostly based on networks of expert centres, institutional collaborations and histology-guided dedicated prospective clinical trials. Nevertheless, although a few trials positioned new standards, new clinical advances are urgently required. The current landscape of oncology is evolving quickly, with the aim to build a personalized journey for patients. Improved understanding of the role of cancer biomarkers, further development of molecularly targeted therapies and the standardization of appropriate targeted treatments into treatment guidelines have shifted clinical practice to a more integrated medicine, especially for common cancers [55–57]. As such, current decade should integrate this model to rare cancers, by considering their specificity, notably regarding RCT development and constraints.

Regarding current trials, several strategies are ongoing (Table 1). Apart from histology-driven trials, which allows evaluation of molecules to a given type, novel trial designs seem appropriate for the specific epidemiology of ROT. Of note, apart from basket and umbrella trials, more innovative and adaptable designs such as master and platform ones could be relevant for clinical research [58]. In the context of ROT, the ongoing phase II, biomarker-driven, BOUQUET (NCT04931342) platform study includes all epithelial ROT and assigns treatment based on molecular alterations, irrespective of histology. This trial is designed with the aim to accelerate the development of biomarker-driven therapies by identifying early signals and establishing proof-of-concept clinical data in patients with recurrent or persistent epithelial ROT. The innovative perspective of this trial, apart from including ROT, is the flexibility in opening new treatment arms (via protocol amendment) as new treatment combinations become available and in closing existing treatment arms that demonstrate minimal clinical activity or unacceptable toxicity. Of note, the first arms opened were ipatasertib and paclitaxel (in case of *PTEN* loss and/or *PIK3CA* or *AKT1* activating mutations), cobimetinib (in case of *BRAF*, *KRAS* or *NRAS* activating mutations and/or *NF1* loss), trastuzumab emtansine (in case of *ERBB2* amplification and/or mutations) and atezolizumab-bevacizumab (in the absence of alterations). The results from this innovative type of trial are highly awaited, both as a proof-of-concept for rare tumours and for future treatment options for ROT.

**Table 1:** Current clinical trials enrolling patients with rare ovarian tumour

NCT number (name; if applicable)	Phase(s)	Histologies	Line(s)	Experimental arm(s)	Comparator arm (if applicable)	Endpoint(s)	Status	Year exp.
NCT04804007 (CTO-IUSCCC-0742)	II	GCT	2^a^	Oral etoposide	Observance	PFS	R	2024
NCT04876456 (CTO-IUSCCC-0752)	II	GCT	≥3	Cabozantinib	–	CBR	R	2024
NCT02834013 (DART)	II	GCT & OCCC^b^	≥2	Nivolumab + ipilumab	–	ORR	R	2023
NCT05026606 (EON)	II	CCC	≥2	Etigilimab + nivolumab	–	ORR	R	2023
NCT03348631 (NRG-GY014)	II	CCC^c^	1–3	Tazemetostat	–	ORR	R	2025
NCT03648489 (DICE)	II	CCC	≥2	TAK228 + paclitaxel	Paclitaxel	PFS	ANR	2023
NCT03355976 (BrUOG 354)	II	CCC	≥2	Nivolumab + ipilumab	Nivolumab (arm closed)	ORR	R	2024
NCT04739800 (NRG-GY023)	II	CCC^d^	2–5	3 arms: cediranib +/− olaparib +/− durvalumab	Paclitaxel or PLD or topotecan	PFS	R	2023
NCT02923739	II	CCC^d^	2–3	Paclitaxel + bevacizumab + emactuzumab	Paclitaxel + bevacizumab	PFS	R	2025
NCT03673124 (GOG-3026)	II	LGSOC	≥2	Letrozole + ribociclib	–	ORR	ANR	2024
NCT04065269 (ATARI)	II	CCC & CS	1–3	AZD6738 + olaparib	AZD6738	ORR	ANR	2023
NCT04625270 (RAMP-201)	II	LGSOC	≥2	VS-6766 + defactinib	VS-6766	ORR	R	2025
NCT03464201 (GREKO III)	II	GCT	All lines	Enzalutamide	Enzalutamide	ORR	ANR	2022
NCT04274426 (MIROVA)	II	CS & CCC^d^	≥2	Mirvetuximab soravtansine + platinum-based regimen	Platinum-based regimen	PFS	R	2023
NCT02839707 (NRG-GY009)	II/III	CCC^e^	2–3	2 arms: PLD + atezolizumab +/− bevacizumab	PLD + bevacizumab	DLT PFS/OS	ANR	2023
NCT03651206 (ROCSAN	II/III	CS	1–3	2 arms (in phase 2): niraparib +/− dostarlimab	Paclitaxel or PLD or topotecan	ORR/OS	R	2025
NCT02429687 (MOGCT-1)	III	GCT	1 - adjuvant	Carboplatin + paclitaxel	BEP	PFS	R	2025
NCT04227522 (MAMOC)	III	CCC^e^	1 - maintenance	Rucaparib	Placebo	PFS	R	2025
NCT04095364 (NRG-GY019)	III	LGSOC	1	Adjuvant carboplatin + paclitaxel then letrozole	Letrozole	PFS	R	2028
NCT04931342 (BOUQUET)	II	eROT^f^	2–5	Biomarker-driven^g^	–	ORR	R	2026

Data were extracted from clinicaltrials.gov (Accessed 01 September 2022). Only phase II and III trials are reported here. ANR, active, not recruiting; BEP, bleomycin + etoposide + cisplatin; CS, carcinosarcoma; CCC, clear cell carcinoma; DLT, dose-limiting toxicity; GCT, germ cell tumour; LGSOC, low-grade serous ovarian carcinoma; ORR, overall response rate; OS, overall survival; PFS, progression-free survival; PLD, pegylated liposomal doxorubicin; R, recruiting; (e)ROT, (epithelial) rare ovarian tumours. Commentaries: ^a^Maintenance after treatment with high-dose chemotherapy and peripheral-blood stem-cell transplant.

b Also includes other ovarian histologies: adenocarcinoma, mucinous adenocarcinoma, small cell carcinoma of the ovary hypercalcaemic type, squamous cell carcinoma and transitional cell carcinoma.

c Restricted to *ARID1A*-mutated cases.

d Also includes mucinous adenocarcinomas and frequent ovarian epithelial tumour types.

e Also includes frequent ovarian epithelial tumour types.

f Persistent or recurrent epithelial ovarian cancer (high-grade serous/endometrioid histologies are excluded).

g Please refer to the text for more detailed information regarding treatment arms for the BOUQUET trial.

## CONCLUSION

The recent few years came with promising and substantial results regarding ROT, starting from the ‘one size fits all’ paradigm towards specific histology-driven therapies. On the basis of biomarker-driven RCT (e.g. basket/umbrella and more recently platform trials), current clinical research could lead in upcoming years to more personalized and efficient management of ROT, supported by national and international consortia and academic research.

## Acknowledgements

*None.*

### Financial support and sponsorship

*None.*

### Conflicts of interest

*S.Q., M.B. and C.L. declare no conflicts of interest. I.R.C. declares the following disclosures: Abbvie, Agenus, Advaxis, BMS, PharmaMar, Genmab, Pfizer, AstraZeneca, Roche, GSK, MSD, Deciphera, Mersena, Merck Sereno, Novartis, Amgen, Tesaro and Clovis; honoraria (institution) from GSK, MSD, Roche and BMS; advisory/consulting fees from Abbvie, Agenus, Advaxis, BMS, PharmaMar, Genmab, Pfizer, AstraZeneca, Roche/Genentech, GSK, MSD, Deciphera, Mersena, Merck Sereno, Novartis, Amgen, Tesaro and Clovis; research grant/funding (self) from MSD, Roche and BMS; research grant/funding (institution) from MSD, Roche, BMS, Novartis, Astra Zeneca and Merck Sereno; and travel support from Roche and AstraZeneca and GSK.*

## REFERENCES AND RECOMMENDED READING

Papers of particular interest, published within the annual period of review, have been highlighted as:

▪ of special interest
▪▪ of outstanding interest
